# Private-Sector Readmissions for Inpatient Surgery in Veterans Health Administration Hospitals

**DOI:** 10.1001/jamanetworkopen.2024.52056

**Published:** 2024-12-26

**Authors:** Mary Vaughan Sarrazin, Yubo Gao, Carly A. Jacobs, Michael A. Jacobs, Susanne Schmidt, Heather Davila, Katherine Hadlandsmyth, Andrea L. Strayer, John Cashy, George Wehby, Paula K. Shireman, Daniel E. Hall

**Affiliations:** 1 Center for Access and Delivery Research and Evaluation, Iowa City Veterans Affairs Medical Center, Iowa City; 2 Department of Internal Medicine, Carver College of Medicine, The University of Iowa, Iowa City; 3 Center for Health Equity Research and Promotion, VA Pittsburgh Healthcare System, Pittsburgh, Pennsylvania; 4 Department of Population Health Sciences, University of Texas Health, San Antonio; 5 Department of Anesthesia, Carver College of Medicine, The University of Iowa, Iowa City; 6 Department of Neurosurgery, The University of Iowa, Iowa City; 7 Department of Health Management & Policy, College of Public Health, The University of Iowa, Iowa City; 8 Departments of Medical Physiology and Primary Care & Rural Medicine, College of Medicine, Texas A&M University, Bryan; 9 Department of Surgery, University of Pittsburgh School of Medicine, Pittsburgh, Pennsylvania; 10 Geriatric Research Education and Clinical Center, VA Pittsburgh Healthcare System, Pittsburgh, Pennsylvania; 11 Wolff Center at University of Pittsburgh Medical Center, Pittsburgh, Pennsylvania

## Abstract

**Importance:**

The Veterans Health Administration (VHA) reports multiple indicators of hospital surgical performance, including hospital risk-standardized 30-day readmission rates (RSRRs). Currently, most routinely reported measures do not include readmissions that occur outside VHA hospitals. The impact of readmissions outside the VHA on hospital RSRR is not known.

**Objective:**

To measure the impact of including non-VHA readmissions on VHA hospital performance rankings for 30-day readmission.

**Design, Setting, and Participants:**

This retrospective cohort study included patients aged at least 65 years from 2013 to 2019 from the Veterans Affairs Surgical Quality Improvement Program linked to patient-level data from the VHA and Medicare. Data were limited to patients with VHA and Medicare enrollment during the year prior to surgery. Data were analyzed from November 2023 through July 2024.

**Main Outcomes and Measures:**

The main outcome was readmissions to acute care VHA or non-VHA hospitals within 30 days of discharge. VHA hospital–level RSRRs were estimated using separate generalized linear mixed-effects risk adjustment models that alternatively included VHA-only or VHA plus non-VHA readmissions. VHA hospitals were then stratified into quintiles based on RSRRs derived using VHA-only or VHA plus non-VHA readmissions. Changes in hospital performance quintiles with the addition of non-VHA readmissions were calculated, and characteristics of VHA hospitals most impacted by including non-VHA readmissions were evaluated.

**Results:**

The eligible cohort included 108 265 patients (mean [SD] age, 72.2 [6.5] years; 105 661 [97.6%] male) who underwent surgery in 104 VHA hospitals. The combined readmission rate was 14.0%. The proportion of readmissions occurring outside the VHA ranged from 0% to 55.3% across the 104 VHA hospitals (median, 20.9%). Using VHA and non-VHA readmissions, 24 VHA hospitals (23.1%) improved performance and 23 hospitals (22.1%) worsened performance, defined as a decrease or increase, respectively, of 1 or more RSRR quintiles. Improvements in hospital performance rank were associated with larger surgical volume (−7.48; 95% CI, −11.33 to 03.64; *P* < .001), urban location, greater surgical complexity (−9.86; 95% CI, −16.61 to −3.11; *P* = .005), and lower proportion of readmissions outside the VHA (−8.15; 95% CI, −12.75 to −3.55; *P* < .001).

**Conclusions and Relevance:**

In this cohort study, VHA hospitals whose readmission performance metric improved by including non-VHA readmissions had higher patient volume, higher complexity, and lower proportion of care outside the VHA. Thus, improving continuity of care may have a paradoxical effect of worsening VHA performance metrics.

## Key Points

**Question:**

Are Veterans Health Administration (VHA) hospital readmission performance metrics impacted by including readmissions to hospitals outside the VHA?

**Findings:**

In this cohort study including 108 265 patients who underwent surgery at 104 VHA hospitals, nearly half (45%) of VHA hospitals changed performance rank when non-VHA readmissions were included in 30-day readmissions. VHA hospitals with higher volume and complexity had lower rates of non-VHA readmissions, leading to improved relative readmission metrics when VHA plus non-VHA readmissions were included.

**Meaning:**

These findings suggest that not accounting for readmissions occurring in non-VHA hospitals may distort hospital performance metrics to the detriment of VHA hospitals with better continuity of care and higher patient volumes and surgical complexity.

## Teaser

This cohort study examines the impact of including non-Veterans Health Administration (VHA) readmission data on VHA hospital performance rankings for 30-day readmission.

## Introduction

Veterans Health Administration (VHA) enrollees often receive care from non-VHA clinicians, typically through Medicare or other insurers.^1,2^ More than 90% of VHA enrollees aged 65 years or older are also Medicare beneficiaries,^3^ with 27.7% receiving health services from community health care practitioners and facilities.^4^ The Care in the Community (CITC) program has significantly expanded veterans’ access to these practitioners.^5^ Initially available on a case-by-case basis, the use of community practitioners through the CITC program has increased from 1.3 million veterans in 2014 to 2.3 million in 2021. Community-based care now constitutes nearly 20% of the VHA clinical budget.^6^

Utilization of hospital services outside the VHA raises considerations for evaluating VHA hospital performance. Readmissions are a commonly used measure of health care quality, particularly in surgery. While the utility of readmissions as a quality metric has been questioned,^7,8^ readmissions as a performance metric are nonetheless used in hospital assessments and may be tied to penalties.^9^ The VHA’s National Surgery Office (NSO) issues quarterly performance reports that incorporate risk-adjusted assessments of hospital readmissions after inpatient operations.^10^ The NSO intervenes to improve quality in facilities with higher-than-expected readmissions. However, the NSO only captures readmissions to VHA facilities; readmissions to private sector facilities are not encompassed in their metrics.

Given the increasing use of community care among veterans, we analyzed how readmissions outside the VHA impact 30-day readmission performance measures. We ascertained changes in hospital-level risk-standardized readmission rates (RSRRs) using VHA readmissions only or VHA plus non-VHA readmissions in a cohort of older veterans. Additionally, we examined whether presurgical care fragmentation moderates the impact of non-VHA readmissions on RSRRs. We hypothesized that including non-VHA readmissions alters hospital RSRR rankings and readmission metrics improve for VHA hospitals with better continuity of care when non-VHA readmissions are included.

## Methods

This cohort study was determined to be exempt from approval and informed consent by the Department of Veterans Affairs (VA) Central Institutional Review Board because it involves only data collected for the purpose of routine health care operations. The study is reported following the Strengthening the Reporting of Observational Studies in Epidemiology (STROBE) reporting guideline

### Design, Setting, and Population

This retrospective cohort study used a national sample of veterans aged 65 years and older in the VA Surgical Quality Improvement Program (VASQIP) during 2013 to 2019 undergoing surgical procedures in VHA hospitals. VASQIP is an audited quality assessment database containing nurse-abstracted data on VHA surgical patients, using methods described elsewhere.^11^ Admissions with length of stay less than 1 day or greater than 30 days were excluded, as these were either not major surgical procedures or care-intensive outliers, respectively. Patients undergoing surgery within 30 days of a preceding surgery were also excluded to ensure independence between admissions.

### Data Sources

The VASQIP cohort was linked to race, sex, and diagnosis code data from the VHA Corporate Data Warehouse electronic health records; Medicare claims from Centers for Medicare & Medicaid Services (CMS); claims from the VHA fee-based and Program Integrity Tool files, which include claims for services provided by non-VHA facilities and clinicians and paid by the VHA; VHA hospital characteristics from the VA Office of Productivity, Efficiency and Staffing, VA Site Tracking System, and the American Hospital Association Survey.^12^ We further excluded admissions where patients lacked essential clinical or procedure-specific data (eg, operative complexity score), had no VHA care in the 2 years preceding surgery, or were enrolled in Medicare managed care or not in Medicare Fee-for-Service during the year before surgery (to ensure complete data on preexisting conditions, care fragmentation, and non-VHA readmissions). Patients who died during hospitalization or were transferred to another acute care facility were also excluded (patients who died after discharge were retained). Additionally, hospitals with fewer than 20 operations in VASQIP were excluded, as low-volume hospitals are excluded from performance assessments. Complete cohort exclusions are provided in eFigure 1 in Supplement 1.

### Variables

We identified all-cause readmissions as clinical events requiring acute hospitalization within 30 days of discharge from the surgical episode. Readmissions alternatively included returns to a VHA acute inpatient setting at least 1 day and up to 30 days after discharge or returns to a VHA or non-VHA acute inpatient setting at least 1 day and up to 30 days after discharge. Readmissions to VHA hospitals were identified in Corporate Data Warehouse inpatient files using bed section to distinguish acute settings, consistent with VASQIP. Readmissions to non-VHA hospitals were identified in VHA fee-based and Program Integrity Tool claims and CMS inpatient files, using hospital type codes to distinguish acute short-term hospitals.

Clinical factors associated with readmission included frailty (Risk Analysis Index^13^ score), comorbidity (Gagne^14^ score, identified using diagnoses from the 12 months preceding admissions), surgical-induced physiologic stress (expanded Operative Stress Score^15,16,17^ ranking procedures from 1, indicating very low stress, to 5, very high stress), primary surgeon specialty, demographics (age, sex), surgery status (elective, urgent, or emergent), and presentation with preoperative acute serious conditions,^18^ including preoperative indicators of ventilator use, pneumonia, coma, sepsis, septic shock, large blood transfusions, acute kidney failure not requiring dialysis, and kidney failure requiring dialysis within 2 weeks of surgery. We also measured care fragmentation, operationalized as the proportion of days receiving non-VHA care during the 12 months preceding surgical admission, relative to all days of care across data sources.^19^

VHA hospital characteristics included number of acute beds, intensive care unit complexity level (ranging from 1 to 4, with 1 indicating most complex), NSO indicator of hospitals capable of high-complexity operations, hospital patient risk rank (determined by Clinical Classification Software Risk Score^20^ aggregated by facility), rural hospital indicator (identified using Rural-Urban Commuting Area codes^21^ assigned to hospital geographic coordinates), hospital teaching status based on membership in the Council of Teaching Hospitals, mean patient fragmentation score, and percentage of surgical patients with no care received outside the VHA during the year prior to surgery.

### Statistical Analysis

We first examined frequency of readmissions by patient characteristics overall and by type of hospital where the readmission occurred (ie, VHA vs non-VHA). Second, we compared readmission rates and characteristics of hospitals stratified by the proportion of readmissions that occur in non-VHA hospitals (>50th percentile vs ≤50th percentile when ranking VHA hospitals by the proportion of readmissions outside VHA). Patient and hospital characteristics were compared using χ^2^ tests for categorical variables and *t* tests for continuous variables.

Subsequently, we assessed hospital-level RSRRs using statistical methods used by CMS to estimate ratios of predicted to expected (P:E) readmissions.^22^ Hospital P:Es and RSRRs were estimated using generalized linear mixed logistic regression models with previously described clinical factors associated with 30-day readmission and hospital random intercepts. Initially, the dependent variable was defined using readmission to VHA hospitals only. Each hospital’s expected readmission rate was estimated by applying regression coefficients associated with risk factors to the hospital’s VASQIP patient population and calculating the expected readmission rate for the hospital. Each hospital’s predicted readmission rate was obtained by adding the best linear unbiased predictor from the random intercepts model, which represents the hospital-specific effect. The P:E readmissions for a VHA hospital represents the performance of the hospital relative to the mean VHA hospital, taking into account their patient population. A lower P:E indicates a lower-than-expected readmission rate, and a higher ratio indicates higher-than-expected readmissions. The RSRR for each hospital was calculated by multiplying the hospital-specific P:E by the national readmission rate. We then repeated the process of estimating hospital P:E, redefining the dependent variable to include readmissions that occurred in either a VHA or non-VHA hospital.

To examine changes in readmission performance rank with the addition of non-VHA readmissions, we first stratified hospitals into quintiles based on P:Es derived from VHA readmissions alone vs VHA plus non-VHA readmissions. Quintiles were ranked from lowest to highest performing. We assigned each hospital a score reflecting the change in quintile when non-VHA readmissions were included in P:E estimation, relative to the quintile without non-VHA readmissions: +1 point for each upward quintile shift (signifying worsening performance rank) and −1 point for each downward quintile shift (signifying improved performance rank). Second, we used a bootstrap process to identify statistical outlier hospitals based on VHA-only or VHA plus non-VHA readmissions. Specifically, we estimated 1000 sets of hospital P:Es by repeatedly modeling cohort samples selected with replacement. We calculated 95% CIs around P:Es to identify hospitals with CIs entirely above or below 1.0.^22^

Subsequently, we examined the association between hospital characteristics and the change in hospital P:E rank when adding non-VHA readmissions using multivariable linear regression in which the dependent variable was the change in hospital P:E rank when non-VHA readmissions were added to P:E estimation, relative to the rank without non-VHA readmissions. Collinearity among candidate hospital characteristics was assessed using a variance inflation factor criterion greater than 10 and bivariable correlation greater than 0.6; when 2 or more hospital characteristics were significantly correlated, the variable that maximized model fit was retained.

Secondary analyses assessed whether presurgical care fragmentation moderates the effect of adding non-VHA readmissions to P:E and RSRR estimation. Specifically, care fragmentation was included in risk adjustment models used to estimate hospital P:Es. Changes in hospital P:E, quintile rank, outlier status, and association between hospital characteristics and change in P:E rank were reassessed. All *P* values were 2-tailed and considered significant at *P* < .05. Analyses were performed using SAS software version 9.4 (SAS Institute) from November 2023 through July 2024.

## Results

### Population Demographics

The analytic cohort contained 108 265 patients (mean [SD] age, 72.2 [6.2] years; 105 661 [97.6%] male) who underwent inpatient surgery at 104 VHA hospitals (Table 1; eFigure 1 in Supplement 1). Half of surgical procedures were moderately stressful (Operative Stress Score 3: 55 880 procedures [51.6%]), followed by low stress procedures (Operative Stress Score 1-2: 36 779 procedures [34.0%]). Most procedures were elective, while 20 167 procedures (18.6%) were urgent and 8595 procedures (7.9%) were emergent. The combined readmission rate was 14.0%, with 3137 readmissions (20.7%) occurring in non-VHA hospitals. Most non-VHA readmissions (2142 readmissions [68.3%]) were paid by CMS; the remainder (995 readmissions [31.7%]) were paid by VA community purchased care programs.

**Table 1.** Patient Characteristics by VHA Readmission and Non-VHA Readmission

Characteristic	All patients, No.	Any readmission, No. (% of total)	Readmissions, No. (% of readmissions)		*P* value
			VHA	Non-VHA	
Overall	108 265	15 167 (15.0)	12 030 (79.3)	3137 (20.7)	NA
Demographics					
Age, y					
Mean (SD)	72.3 (6.5)	73.1 (6.7)	72.9 (6.6)	74.1 (7.2)	<.001
65-74	78 569	10 281 (13.1)	8354 (81.3)	1927 (18.7)	<.001
75-84	22 976	3666 (16.0)	2791 (76.1)	875 (23.9)	
85-94	6374	1148 (18.0)	833 (72.6)	315 (27.4)	
≥95	346	72 (20.8)	52 (72.2)	20 (27.8)	
Sex					
Male	105 661	14 872 (14.1)	11 792 (79.3)	3080 (20.7)	.56
Female	2604	295 (11.3)	238 (80.7)	57 (19.3)	
Comorbidity					
Gagne Score					
0-1	38 182	3073 (8.1)	2465 (80.2)	608 (19.8)	.17
2-3	27 727	3368 (12.2)	2686 (79.8)	682 (20.3)	
4-6	23 623	4159 (17.6)	3292 (79.2)	867 (20.9)	
7-10	14 604	3301 (22.6)	2613 (79.2)	688 (20.8)	
11 or more	4129	1266 (30.7)	974 (76.9)	292 (23.1)	
Frailty^a^					
0 to <25	32 063	3174 (9.9)	2626 (82.7)	548 (17.3)	<.001
25 to <30	39 734	4547 (11.4)	3634 (79.9)	913 (20.1)	
30 to <35	19 159	3213 (16.8)	2503 (77.9)	710 (22.1)	
35 to <40	9352	2065 (22.1)	1616 (78.3)	449 (21.7)	
40 to <45	4283	1104 (25.8)	838 (75.9)	266 (24.1)	
≥45	3674	1064 (29.0)	813 (76.4)	251 (23.6)	
PASC	4279	1099 (25.7)	879 (80.0)	220 (20.0)	.57
Surgery characteristics					
Surgical specialty					
General surgery	25 924	3943 (15.2)	3231 (81.9)	712 (18.1)	<.001
Neurology	6534	832 (12.7)	632 (76.0)	200 (24.0)	
Thoracic	6335	841 (13.3)	691 (82.2)	150 (17.8)	
Urology	9590	1370 (14.3)	1080 (78.8)	290 (21.2)	
Peripheral vascular	18 762	3850 (25.4)	3138 (81.5)	712 (18.5)	
Other	41 120	4331 (10.5)	3258 (75.2)	1073 (24.8)	
Operative-induced stress^b^					
1 (Very low)	1720	316 (18.4)	256 (81.0)	60 (19.0)	<.001
2 (Low)	35 059	4242 (12.1)	3313 (78.1)	929 (21.9)	
3 (Medium)	55 880	7974 (14.3)	6265 (78.6)	1709 (21.4)	
4 (High)	13 962	2311 (16.6)	1917 (83.0)	394 (17.1)	
5 (Very high)	1644	324 (19.7)	279 (86.1)	45 (13.9)	
Surgery status					
Elective	79 503	9489 (11.9)	7543 (79.5)	1946 (20.5)	.34
Urgent	20 167	3936 (19.5)	3092 (78.6)	844 (21.4)	
Emergent	8595	1742 (20.3)	1395 (80.1)	347 (19.9)	
Care fragmentation					
Care days with non-VHA care, mean (SD), %	22.1 (33.1)	24.7 (33.6)	22.6 (32.6)	32.7 (35.8)	<.001
Received all care in VHA	59 080	7316 (12.4)	6147 (84.0)	1169 (16.0)	NA
≤50% Of care received outside VHA	28 167	4610 (16.4)	3571 (77.5)	1039 (22.5)	NA
>50% Of care received outside VHA	21 018	3241 (15.4)	2312 (71.3)	929 (28.7)	NA

Abbreviations: NA, not applicable; PASC, preoperative acute serious condition; VHA, Veterans Health Administration.

^a^ Assessed using Risk Analysis Index score.

^b^ Assessed using the Operative Stress Score.

Table 2 compares readmission statistics and characteristics of VHA hospitals stratified by the proportion of readmissions that occurred in non-VHA hospitals. The mean (SD) readmission rate across all 104 VHA hospitals was 13.7% (2.9), and the mean (SD) proportion of readmissions that occurred outside the VHA was 22.3% (8.6). Of those, the mean proportion paid by VHA’s community purchased care programs across hospitals was 31.5% (16.8). The total readmission rate did not differ between hospitals with a relatively low vs relatively high proportions of non-VHA readmissions (mean [SD] rate, 13.9% [2.5] vs 13.5% [3.2]; *P* = .47), although the proportion of readmissions occurring in non-VHA hospitals differed (16.1% [4.3] vs 28.6% [7.1]; *P* < .001). Hospitals with a higher proportion of non-VHA readmissions tended to have lower surgical volumes (mean [SD], 876 [553] vs 1206 [500] procedures; *P* = .002), were less likely to have a level 1 intensive care unit (20 hospitals [38.5%] vs 32 hospitals [61.5%]; *P* = .02), were less likely to be designated as capable of high operative complexity (26 hospitals [50.0%] vs 41 hospitals [78.9%]; *P* = .002), had lower patient risk rank (mean [SD] rank, 58 [29] vs 46 [30]; *P* = .05), and had fewer veterans who received 100% of their care in the prior 365 days through the VHA (54.7% [6.9] vs 58.7% [6.4]; *P* = .003).

**Table 2.** Hospital Characteristics Overall and Ranked by Percentage of Readmissions That Occur Outside the VHA

Characteristic	Mean (SD), %			*P* value
	All hospitals (N = 104)	Percentile of non-VHA readmissions,		
		<50th (n = 52)	≥50th (n = 52)	
Combined readmission	13.7 (2.9)	13.9 (2.5)	13.5 (3.2)	.47
VHA-only readmission	10.7 (2.6)	11.6 (2.1)	9.7 (2.7)	<.001
Non-VHA readmission	3.0 (1.2)	2.2 (0.7)	3.8 (1.1)	<.001
Readmissions occurring outside VHA	22.3 (8.6)	16.1 (4.3)	28.6 (7.1)	<.001
Non-VHA care through VHA-purchased community care	31.5 (16.8)	31.7 (17.8)	32.4 (15.8)	.60
Hospital characteristics				
VASQIP 2013-2019 patients per hospital, mean (SD), No.	1041 (550)	1206 (500)	876 (553)	.002
Bed size, mean (SD), No.	254 (227)	272 (189)	237 (260)	.49
ICU level 1 (most complex)	52 (50.0)	32 (61.5)	20 (38.5)	.02
Highest NSO operative complexity	67 (64.4)	41 (78.9)	26 (50.0)	.002
Rural hospital	7 (6.7)	2 (3.9)	5 (9.6)	.24
COTH hospital	27 (26.0)	11 (21.2)	16 (30.8)	.27
Patient risk rank^a^	52 (29)	58 (29)	46 (30)	.05
Hospital mean patient fragmentation				
Percentage of days of care with non-VHA care	22.5 (6.6)	21.2 (6.6)	23.7 (6.8)	.07
Patients with all prior year care received in VHA	56.7 (6.3)	58.7 (6.4)	54.7 (6.9)	.003

Abbreviations: COTH, Council of Teaching Hospitals; ICU, intensive care unit; NSO, National Surgery Office; VASQIP, Department of Veterans Affairs Surgical Quality Improvement Program; VHA, Veterans Health Administration.

^a^ Patient risk rank reported by Department of Veterans Affairs Office of Productivity, Efficiency and Staffing was reversed so that higher rank indicates higher patient risk.

### Risk Adjustment With and Without Non-VHA Readmissions

Risk adjustment models with VHA readmissions only or VHA plus non-VHA readmissions as the dependent variable are presented in eTable 1 in Supplement 1. Discrimination for both models was acceptable (C statistic = 0.67). Odds ratios (ORs) associated with clinical risk factors were generally similar regardless of whether the outcome included solely VHA readmissions or VHA plus non-VHA readmissions. However, ORs associated with the RAI frailty score and Gagne comorbidity score were larger in the model that included non-VHA readmissions, and the difference increased with higher frailty and comorbidity.

### Hospital Quintile Rankings and Outlier Status

**Figure 1.**
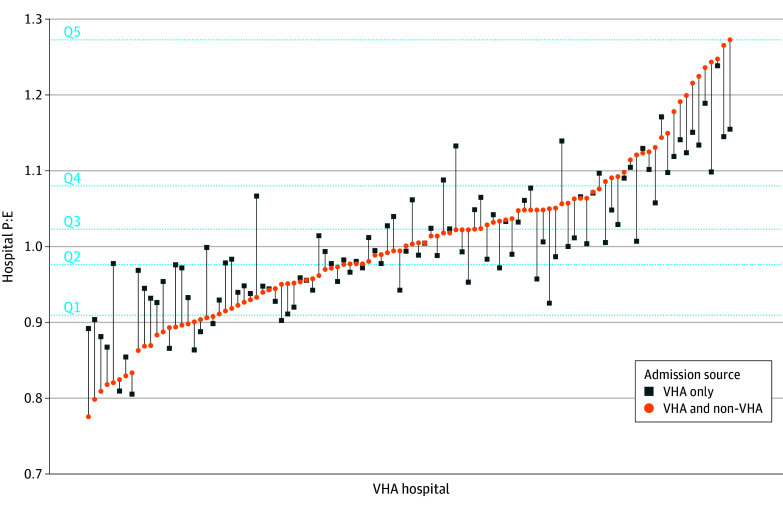
Hospital Predicted-to-Expected Ratios (P:Es) for Readmissions Based on Veterans Health Administration (VHA) Readmissions Only vs VHA Plus Non-VHA Readmissions Hospital P:Es are ranked from lowest (best performing) to highest (worst performing) based on model that defines outcome using VHA readmissions only, vs VHA plus non-VHA readmissions. Q indicates quintile.

**Figure 2.**
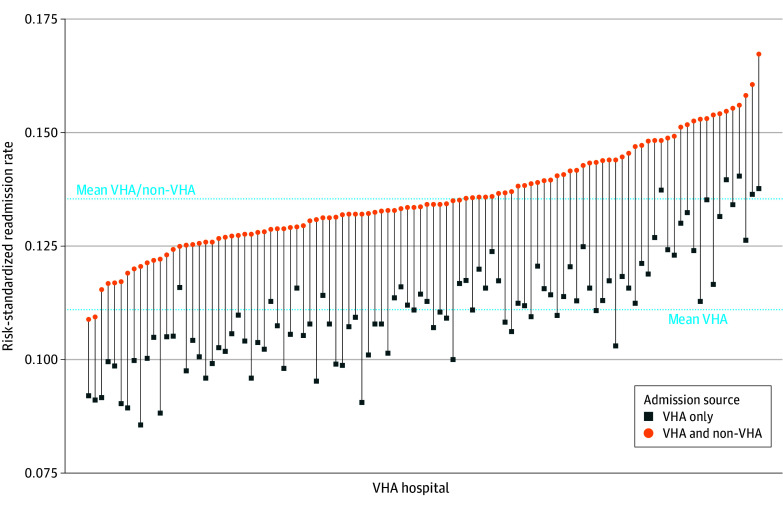
Changes in Hospital Risk-Standardized Readmission Rate Based on Veterans Health Administration (VHA) Readmissions Only vs VHA Plus Non-VHA Readmissions The change in risk-standardized readmission rate with the addition of non-VHA readmissions is illustrated with vertical lines. The mean VHA-only and the mean VHA plus non-VHA readmission rates are illustrated as horizontal lines.

Figure 1 shows the distribution of hospital P:Es when the outcome includes VHA readmissions only or VHA plus non-VHA readmissions. A decrease in hospital P:E suggests improved estimated performance (ie, lower risk-adjusted readmission), while an increase in P:E suggests worse performance. Of 104 hospitals assessed, 57 (54.8%) did not change P:E quintile, 24 (23.1%) decreased by 1 or more quintiles, and 23 (22.1%) increased by 1 or more quintiles. Figure 2 shows the distribution of RSRR based on VHA-only and VHA plus non-VHA readmissions and change in RSRR with the addition of non-VHA readmissions. RSRRs increased for all hospitals with the addition of non-VHA readmissions, although the magnitude of increase varied. Finally, the number of hospitals classified as statistical outliers when adding non-VHA readmissions decreased from 9 to 5 high-readmission outliers and from 4 to 2 low-readmission outliers.

Table 3 shows associations between hospital characteristics and the change in P:E rank with the addition of non-VHA readmissions to the outcome. In bivariable regressions, hospital P:E rank decreased significantly as surgical patient volume increased (P:E rank change, −7.48; 95% CI, −11.33 to 03.64; *P* < .001), in hospitals capable of complex surgeries (P:E rank change, −9.86; 95% CI, −16.61 to −3.11; *P* = .005) and intensive care (P:E rank change, −10.12; 95% CI, −16.54 to −3.69; *P* = .003), and in hospitals with a greater percentage of patients receiving all care through the VHA (P:E rank change, −8.15; 95% CI, −12.75 to −3.55; *P* < .001).

**Table 3.** Linear Regression With Change in Hospital P:E Rank as Dependent Variable

Variable	Univariable analyses		Multivariable analysis	
	Coefficient (95% CI)	*P* value	Coefficient (95% CI)	*P* value
Hospital beds, per 1000 beds	−1.22 (−2.69 to 0.26)	.11	−0.75 (−2.26 to 0.75)	.33
No. of patients, per 10 000 patients	−7.48 (−11.33 to −3.64)	<.001	NA^a^	NA^a^
Complex surgery capable	−9.86 (−16.61 to −3.11)	.005	−3.42 (−11.47 to 4.64)	.41
ICU level 1 (most complex)	−10.12 (−16.54 to −3.69)	.003	NA^a^	NA^a^
COTH	0.95 (−6.71 to 8.61)	.81	6.95 (−0.65 to 14.56)	.08
Rural hospital	10.88 (−2.37 to 24.12)	.11	6.90 (−5.97 to 19.77)	.30
Patient risk rank	1.80 (0.74 to 2.86)	.001	1.34 (0.13 to 2.55)	.03
Percentage of patients with 100% VHA care, per 10%	−8.15 (−12.75 to −3.55)	<.001	−5.03 (−0.03 to −10.03)	.05
Percentage of mean presurgical care days outside VHA, per 1 percentage point	3.83 (−1.09 to 8.75)	.13	NA^a^	NA^a^

Abbreviations: COTH, Council of Teaching Hospitals; ICU, intensive care unit; NA, not applicable; VHA, Veterans Health Administration.

^a^ Variables not included due to collinearity with other hospital characteristics.

Secondary analysis further controlled for care fragmentation in risk adjustment models used to estimate P:Es. Care fragmentation, defined as receipt of non-VHA care prior to surgery, was not associated with risk of readmission in VHA-only models (OR, 0.97; 95% CI, 0.93-1.01; *P* = .12) but was associated with higher risk in models that included VHA and non-VHA readmissions (OR, 1.10; 95% CI, 1.04-1.07; *P* < .001). After categorizing hospitals into performance quintiles based on P:E, 24 hospitals decreased 1 or more quintiles and 25 hospitals increased 1 or more quintiles (eFigure 2 in Supplement 1). Associations between hospital characteristics and change in P:E rank demonstrated patterns similar to the primary analysis (eTable 2 in Supplement 1). In bootstrap analysis, the number of hospitals identified as high and low P:E outliers was identical to the primary analysis.

## Discussion

In this cohort study, we quantified the impact of private sector readmissions on VHA readmission performance measures using multiple data sources linked to VASQIP data. Overall, 1 of 5 readmissions were to non-VHA hospitals, with the proportion varying across hospitals. Notably, the combined readmission rate was similar between hospitals with relatively low and relatively high proportions of non-VHA readmissions. Nearly half of VHA hospitals changed quintile rank when non-VHA readmissions were included in analysis, with performance improving for 23% of hospitals. In addition, the number of hospitals classified as statistical outliers decreased. VHA hospitals whose rank improved by including non-VHA readmissions tended to be larger and more complex, and served patients less likely to have a history of care outside the VHA. Overall findings were similar in analysis in which readmission risk adjustment models also adjusted for presurgical care fragmentation.

Our finding that 20% of readmissions occurred outside the VHA is consistent with other studies showing that sizable proportions of veterans receive care outside the VHA. The use of community care by veterans has increased substantially since the passage of legislation to improve access to care.^23^ The Veterans Access, Choice and Accountability Act of 2014^24^ and the 2018 John S. McCain III, Daniel K. Akaka, and Samuel R. Johnson VA Maintaining Internal Systems and Strengthening Integrated Outside Networks Act^25^ expanded options for Veterans to receive care through community health care practitioners and facilities paid by the VHA. The VHA budget for community care has increased from 12% in 2014 to 25% of the projected budget in 2024,^6^ In addition, 90% of veterans older than age 65 years are enrolled in Medicare.^3^

Readmission is a high-priority performance metric that is the focus of national efforts to improve quality of care through public reporting,^26,27^ financial incentives,^9^ and oversight.^28^ The increasing use of non-VHA services impacts VHA hospital performance metrics in 2 ways. First, excluding non-VHA services can introduce bias into measures. In our study, hospitals with low non-VHA utilization tended to be unfairly penalized, suggesting that enhancing care continuity might paradoxically lower VHA readmission performance ratings. Conversely, overlooking non-VHA services for certain metrics may penalize hospitals with higher non-VHA utilization (eg, missing outside cancer screenings). Likewise, comparisons among private-sector health care systems may misrepresent performance if use of external services varies. Payer-based assessments, like those reported on CMS Care Compare, encompass all payer-funded services irrespective of location. However, private-sector clinical registries, like the National Surgical Quality Improvement Program, only capture care within a single hospital system. Similarly, VASQIP currently captures care occurring within the VHA.

The second implication is that VHA hospitals have limited influence over the quality of non-VHA services, potentially affecting VHA hospital performance metrics negatively. Poor coordination of postdischarge care, changes in medication, or conflicting self-care instructions can increase readmission risk.^29^ Our study found that presurgical care fragmentation was associated with a 10% higher readmission risk, possibly indicating unmeasured risk or adverse effects of fragmented care. As veterans increasingly access community care, VHA clinicians face greater responsibility for care received outside their facilities. Effective coordination, including information sharing with non-VHA clinicians, is crucial to ensure veterans receive quality care. The VHA’s Office of Integrated Veteran Care, established in October 2021, supports this coordination effort.

### Limitations

This study has some limitations. While our analysis underscores the importance of non-VHA readmissions in evaluating readmission performance, we have not attempted to duplicate current VHA metrics. The VHA uses multiple performance assessment systems, including VHA NSO, the VHA’s Strategic Analytics for Improvement and Learning (SAIL),^30^ and the VA Inpatient Evaluation Center (IPEC), each using varied data sources, patient populations, and statistical approaches to assess hospital readmissions. Unlike administrative data relied on by SAIL and IPEC, both NSO and our study integrate VASQIP, a clinical registry offering enhanced capture and accurate reporting of preoperative conditions.^31^ By merging VASQIP with multiple data sources, we achieve a comprehensive view of postsurgical readmissions beyond VASQIP’s standalone capability. Moreover, SAIL and IPEC focus broadly on hospitalwide and specific medical conditions, respectively, which may not fully reflect surgical readmission performance. A 2024 study by Diaz et al^32^ found significant differences in surgical outcomes among hospitals categorized as high and low quality by CMS Quality Star ratings. This indicates that performance in one clinical area may not reflect performance in others, suggesting that SAIL and IPEC reports might not accurately reflect surgical readmission performance. NSO’s metrics encompass VASQIP data for adults aged 18 years and older, whereas our analysis specifically limits the cohort to patients aged at least 65 years to capture services outside the VHA using Medicare claims. We acknowledge that readmissions to non-VHA facilities may be more prevalent among Medicare-covered patients compared with younger individuals. IPEC evaluates VHA hospitals’ readmission performance for patients aged 65 years and older using combined VHA and Medicare data, facilitating comparisons with community hospitals on CMS Care Compare. We also note likelihood of readmission varies by insurance type^33^ and private sector hospitals face different financial incentives to admit patients than the VHA. The proportion of readmissions that occur outside VHA hospitals may differ for patients with insurance other than Medicare. Additionally, our statistical approach aligns with CMS Care Compare, IPEC, and SAIL by shrinking hospital readmission rates to mean levels, particularly for smaller hospitals. In contrast, NSO compares observed-to-expected readmission rates, although the use of the shrinkage method has been recommended.^34^ Furthermore, we note that the utility of readmissions as a quality metric has been questioned.^7,8^ There is general acknowledgment that factors outside the control of surgical health care practitioners, such as lack of social support, insurance status, homelessness, or health-related postdischarge behaviors, influence the likelihood of readmissions.^33,35,36^ While prior studies suggest that most readmissions after inpatient surgery are likely related to surgical quality, up to one-third are not, and the proportion not directly related to surgical quality varies substantially by surgical procedure.^37^ Moreover, psychosocial factors influencing readmission risk may be exacerbated in VHA hospitals, as veterans are more prone to substance use, mental illness, homelessness, and social isolation than the general population.^38,39,40,41^ Alternative definitions of readmissions have been proposed to better capture issues related to quality of care.^42,43^ Nevertheless, our goal was to understand the influence of non-VHA care on readmission performance using current risk adjustment paradigms. Therefore, we define readmissions in a manner consistent with current performance reports and have not attempted to control for external social risk factors in our analysis.

## Conclusions

The findings of this cohort study demonstrate the importance of considering out-of-system readmissions in assessments following inpatient surgeries. Readmission is a high-priority performance metric receiving national attention in quality improvement efforts. As VHA-purchased care continues to increase, the impact of out-of-system readmissions becomes increasingly significant. Our findings have implications for quality assessment within the VHA and external assessments that rely on incomplete data regarding patient services.

## Notes

Supplement 1. **eFigure 1.** Cohort Flow Diagram **eFigure 2.** Number of Hospitals That Change Performance Quintile When Non-VA Readmissions Are Included in Readmission Outcome **eTable 1.** Odds Ratios for Clinical Variables in Risk Adjustment Model Estimating Risk of 30-Day Readmission, Where Readmission Includes VHA Only Readmissions vs VHA + Non-VHA Readmissions **eTable 2.** Linear Regression With Change in Hospital P/E Rank as Dependent Variable

Supplement 2.
Data Sharing Statement
