# Supplementary material for: Private-Sector Readmissions for Inpatient Surgery in Veterans Health Administration Hospitals
Source: JAMA Netw Open. 2024 Dec 26;7(12):e2452056. doi: 10.1001/jamanetworkopen.2024.52056 (PMC11672159; doi:10.1001/jamanetworkopen.2024.52056)
Supplement: Supplement 1. — eFigure 1. Cohort Flow Diagram eFigure 2. Number of Hospitals That Change Performance Quintile When Non-VA Readmissions Are Included in Readmission Outcome eTable 1. Odds Ratios for Clinical Variables in Risk Adjustment Model Estimating Risk of 30-Day Readmission, Where Readmission Includes VHA Only Readmissions vs VHA + Non-VHA Readmissions eTable 2. Linear Regression With Change in Hospital P/E Rank as Dependent Variable [file jamanetwopen-e2452056-s001.pdf]

Supplemental Online Content

Sarrazin MV, Gao Y, Jacobs CA, et al. Private-sector readmissions for inpatient surgery in Veterans Health Administration hospitals. *JAMA Netw Open*. 2024;7(12):e2452056. doi:10.1001/jamanetworkopen.2024.52056

eFigure 1. Cohort Flow Diagram
eFigure 2. Number of Hospitals That Change Performance Quintile When Non-VA Readmissions Are Included in Readmission Outcome
eTable 1. Odds Ratios for Clinical Variables in Risk Adjustment Model Estimating Risk of 30-Day Readmission, Where Readmission Includes VHA Only Readmissions vs VHA + Non-VHA Readmissions
eTable 2. Linear Regression With Change in Hospital P/E Rank as Dependent Variable

This supplemental material has been provided by the authors to give readers additional information about their work.

© 2024 Sarrazin MV et al. *JAMA Network Open.*

Supplemental Figure 1: Cohort Inclusion/Exclusion

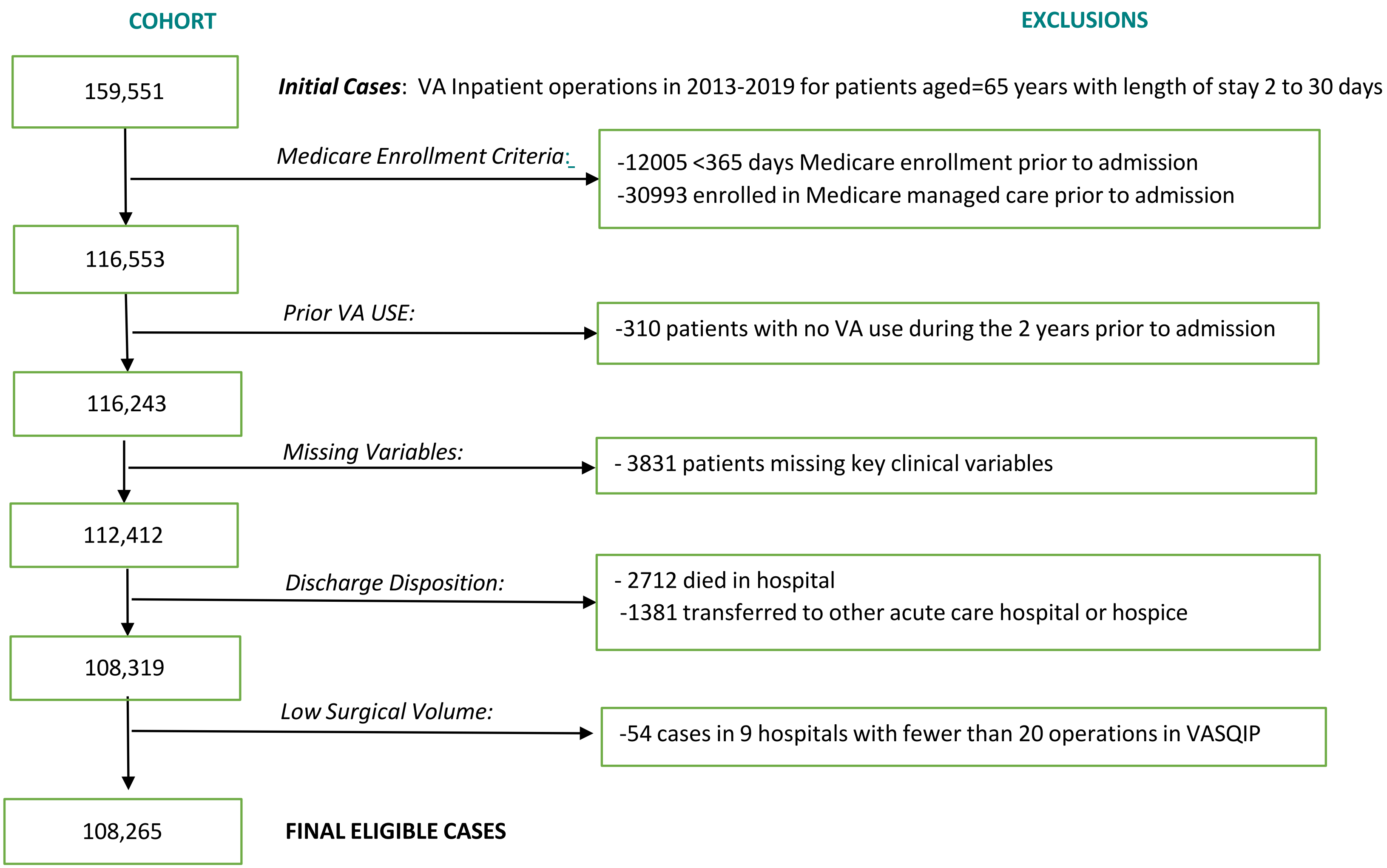

© 2024 Sarrazin MV et al. *JAMA Network Open.*

Supplemental Table 1. Odds Ratios for clinical variables in risk adjustment model estimating risk of 30-day readmission, where readmission includes VHA only readmissions versus VHA + non-VHA readmissions.

**Variable**	**Outcome defined using VHA readmissions only**	**Outcome includes VHA + Non--VHA readmissions**
Age >=85	0.80 (0.74-0.87; p<.001)	0.87 (0.81-0.93; p<.001)
Gagne Comorbidity Score (Ref=0-1)		
2-3	1.35 (1.27-1.43; p<.001)	1.40 (1.32-1.48; p<.001)
4-6	1.79 (1.69-1.90; p<.001)	1.86 (1.77-1.96; p<.001)
7-10	2.13 (1.99-2.27; p<.001)	2.22 (2.09-2.36; p<.001)
11 or more	2.69 (2.45-2.94; p<.001)	2.96 (2.73-3.22; p<.001
RAI-A Frailty Score (ref=<25)		
25-<30	1.03 (0.98-1.09; p=0.23)	1.08 (1.03-1.13; p=.003)
30-<35	1.30 (1.22-1.38; p<.001)	1.40 (1.32-1.48; p<.001)
35-<40	1.54 (1.43-1.65; p<.001)	1.66 (1.55-1.78; p<.001)
40->45	1.65 (1.50-1.82; p<.001)	1.86 (1.71-2.03; p<.001)
45 or higher	1.76 (1.59-1.94; p<.001)	1.97 (1.79-2.16; p<.001)
Surgical Specialty (Ref=General Surgery)		
Neurology	0.96 (0.87-1.06; p=0.41)	1.07 (0.98-1.16; p=0.13)
Thoracic Surgery	0.80 (0.73-0.88; p<.001)	0.83 (0.76-0.90; p<.001)
Urology	1.05 (0.98-1.14; p=0.18)	1.11 (1.04-1.19; p=.003)
Peripheral Vascular	1.42 (1.34-1.50; p<.001)	1.44 (1.37-1.52; p<.001)
All Others	0.79 (0.75-0.84; p<.001)	0.86 (0.82-0.91; p<.001)
Operative Complexity (OSS) (Ref=3)		
OSS 1-2	1.00 (0.96-1.05; p=0.90)	1.00 (0.96-1.04; p=0.89)
OSS 4	1.38 (1.30-1.47; p<.001)	1.34 (1.26-1.42; p<.001)
OSS 5	1.45 (1.27-1.66; p<.001)	1.37 (1.21-1.56; p<.001)
Presentation Acuity		
Case Status (Ref=Elective)		
Urgent	1.35 (1.29-1.42; p<.001)	1.37 (1.31-1.43; p<.001)
Emergent	1.41 (1.32-1.52; p<.001)	1.42 (1.33-1.51; p<.001)
PASC	1.29 (1.18-1.40; p<.001)	1.27 (1.18-1.38; p<.001)

© 2024 Sarrazin MV et al. *JAMA Network Open.*

Supplemental Figure 2.

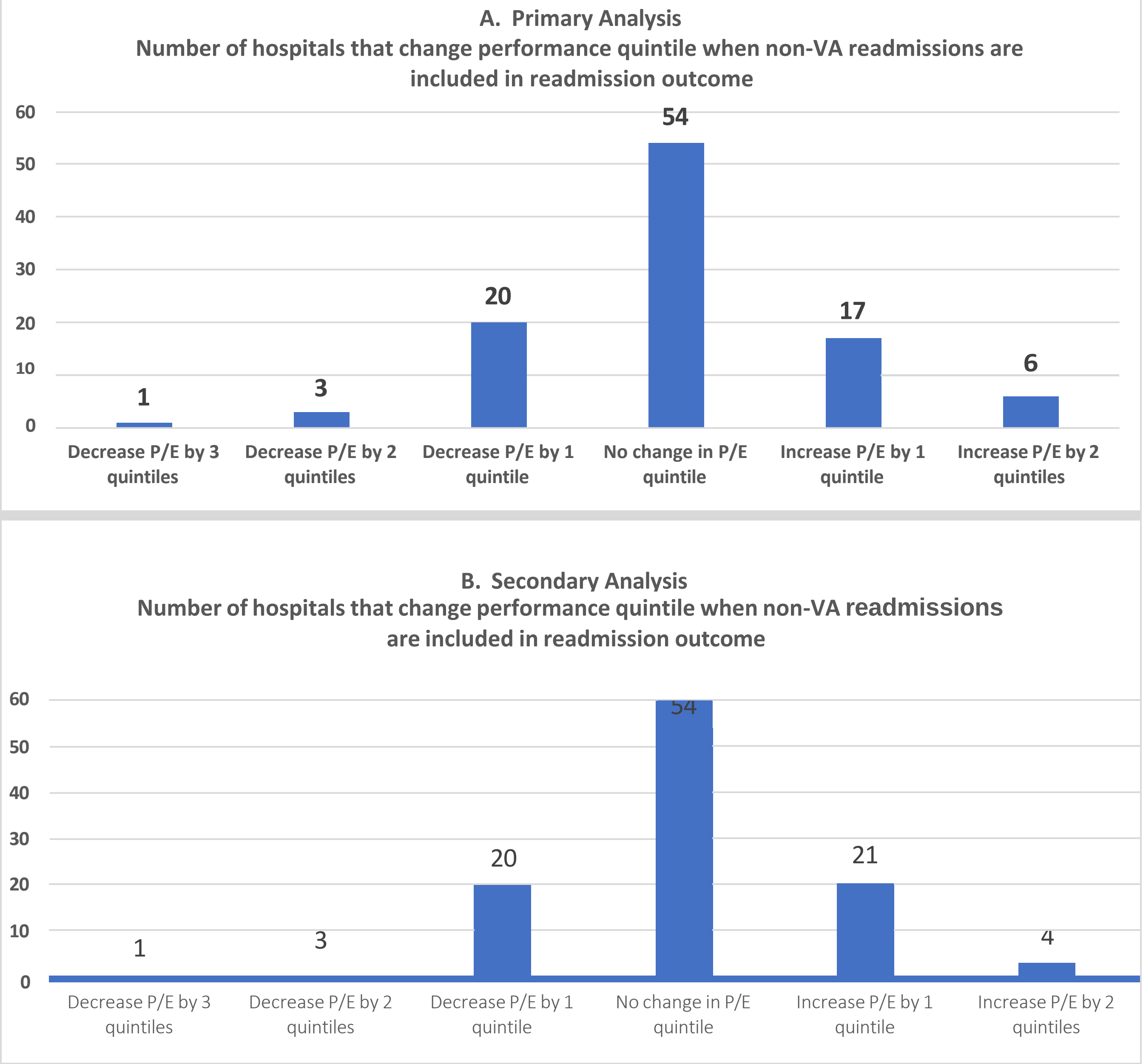

© 2024 Sarrazin MV et al. *JAMA Network Open.*

Supplemental Table 2:
Linear Regression with Change in hospital P/E rank as dependent variable:

Variable Name	Univariable Analyses	Multivariable analysis
	**Coefficient (95% CI; p-value)**	**Coefficient (95% CI, p-value)**
Hospital Beds / 1000	-1.08 (-2.48, 0.32; p=0.13)	-0.75 (-2.22, 0.71; p =0.32)
Number of Patients / 10000	-6.59 (-10.28, -2.91; p<.001)	collinear
Complex Surgery Capable	-8.60 (-15.05, -2.15; p=0.01)	-3.22 (-11.04, 4.60; p=0.42)
ICU Level 1 (Most Complex)	-9.27 (-15.39, -3.15; p=0.004)	collinear
Council of Teaching Hospital	1.15 (-6.12, 8.42; p=0.76)	6.52 (-0.87, 13.90; p=0.09)
Rural Hospital	9.34 (-3.26, 21.94; p=0.15)	5.90 (-6.59, 18.40; p=0.36)
Patient Risk Rank	1.59 (0.57, 2.60; p=0.003)	1.27 (0.10, 2.44; p=0.04)
Percent of patients with 100% VA Care (per 10%)	-6.34 (-10.79, -1.90; p=0.006)	-3.82 (-8.67, 1.03; p=0.18)
Percent pre-surgical care days outside VHA	2.41 (-2.30, 7.11; p=0.32)	collinear

**Variables not included due to collinearity*

© 2024 Sarrazin MV et al. *JAMA Network Open.*
