# Supplementary material for: Private-Sector Readmissions for Inpatient Surgery in Veterans Health Administration Hospitals
Source: JAMA Netw Open. 2024 Dec 26;7(12):e2452056. doi: 10.1001/jamanetworkopen.2024.52056 (PMC11672159; doi:10.1001/jamanetworkopen.2024.52056)
Supplement: Supplement 2. — Data Sharing Statement [file jamanetwopen-e2452056-s002.pdf]

Data Sharing Statement

Sarrazin. Private-Sector Readmissions for Inpatient Surgery in Veterans Health Administration Hospitals. *JAMA Netw Open*. Published December 26, 2024.
doi:10.1001/jamanetworkopen.2024.52056

Data
Data available: No

Additional Information
Explanation for why data not available: VASQIP data is available to VA investigators through the VA National Surgery OFfice with an approved data use agreement.
